# Imaging the Choroid

**DOI:** 10.3390/vision4030038

**Published:** 2020-08-16

**Authors:** Sumit Randhir Singh, Jay Chhablani

**Affiliations:** 1 Jacobs Retina Center at Shiley Eye Institute, University of California, San Diego, La Jolla, CA 92093-0021, USA; sumit.jipmer@gmail.com; 2 Department of Ophthalmology, University of Pittsburgh Eye and Ear Institute, Pittsburgh, PA 15213, USA

**Keywords:** choroid, optical coherence tomography, optical coherence tomography angiography, adaptive optics, laser Doppler holography

The choroid is the most vascular tissue of the eye, sandwiched between sclera and retina, and responsible for blood supply of the outer retina [1]. Its role has been studied and implicated in various chorioretinal disorders. Thickened, hyperpermeable choroid plays an important role in the pathogenesis of pachychoroid spectrum disorders, whereas age-related macular degeneration is characterized by choroidal thinning [2,3]. The traditionally, dye based invasive test such as indocyanine green angiography was the only imaging modality able to provide two-dimensional, dynamic visualization of the choroid [4]. However, inability to provide depth resolution and constraints with test repeatability due to its invasive nature, limited our understanding of the choroid for decades.

The advancements in ocular imaging especially over the last two decades have resulted in a significant increase in the literature focused on choroid [5]. With the modifications in optical coherence tomography (OCT) such as inclusion of long infrared waves for scan acquisition as employed in swept-source OCT or shifting of zero delay line towards the choroid in enhanced depth imaging, identification of choroidoscleral interface and the choroidal layers is possible [6,7]. Subsequently, advanced models helped to create a 3-dimensional reconstruction of choroid [5]. A list of descriptors can be used to characterize unique choroidal variables: choroidal thickness, volume, vessel layer thickness, vascularity index, hyper-reflective dots [5]. Addition of OCT angiography (OCTA) is helpful in visualization and quantification of choroidal vasculature especially, the choriocapillaris (CC) layer [8]. The correction of ocular aberrations using adaptive optics (AO) and its combination with OCT and OCTA provide high-resolution images to delineate retinal pigment epithelium (RPE) from CC and assessment of CC diameter and density [9,10,11]. Recently, laser Doppler holography has been shown to provide high-resolution images of the choroidal vessels with differentiation of both arterial and venous vasculature based on the blood flow dynamics [12].

The wealth of information provided by these imaging techniques has enhanced our understanding of the state of the choroid in both health and disease. In this Special Issue, we focus on the various innovative techniques of “*imaging the choroid*”.
